# Gender Differences in Forgiveness and its Affective Correlates

**DOI:** 10.1007/s10943-021-01369-5

**Published:** 2021-08-06

**Authors:** Kinga Kaleta, Justyna Mróz

**Affiliations:** Department of Psychology, The Jan Kochanowski University, ul. Krakowska 11, 25-029 Kielce, Poland

## Abstract

Although women are believed to be more forgiving than men, the results of many studies comparing women with men vary. Moreover, little is known about unique correlates or differential patterns of experiencing forgiveness by gender. In the present study, we compared men and women in terms of their level of dispositional forgiveness and its emotional correlates, namely positive and negative affect, anxiety, and emotional control. The sample consisted of 625 individuals aged 19–69, of whom 478 (76.5%) were women and 147 (23.5%) were men. Polish versions of the Heartland Forgiveness Scale (HFS), the Positive and Negative Affect Schedule (PANAS), the Courtauld Emotional Control Scale (CECS), and the State-Trait Anxiety Inventory (STAI) were used. Men showed a higher level of general forgiveness and greater willingness to overcome unforgiveness than women, but there was no significant difference in positive facets of the disposition to forgive. In both genders negative affect, anxiety, and control of anger and of depression were negatively related to dimensions of dispositional forgiveness, and positive affect was positively associated with forgiveness. In females control of anxiety was negatively and in males it was positively related to facets of forgiveness. Gender moderated a number of links between affective traits and forgiveness of self and of situations beyond control, but not forgiveness of others.

## Introduction

Women are generally believed to be more forgiving than men, due to their personality traits such as agreeableness and empathy, and their valuing relationships (Miller et al., 2008). In a meta-analytic review of 70 studies with 15,731 individuals, Miller et al. (2008) found a gender difference in forgiveness with small to moderate effect size, showing greater willingness to forgive among female respondents. On the other hand, Fehr et al. (2010) who conducted meta-analyses across 53 studies with the total number of 8,366 participants, found no relationships between gender and forgiveness. There are many reasons for this discrepancy between findings reported by different researchers, including methodological approach, but the main one is the fact that majority of referenced studies were not aimed at comparing men and women in terms of multidimensional forgiveness and the way they experience this phenomenon. Also, very few attempts have been made to find unique correlates or differential patterns of experiencing forgiveness by genders (e.g., Toussaint & Webb, 2005). Thus, in the present study we directly compare males and females in terms of their level of dispositional forgiveness and its emotional correlates, namely positive and negative affect, anxiety, and emotional control, and we examine the moderating role of gender in the relationships between emotions and forgiveness.

### Forgiveness—Disposition and Gender Differences

Most researchers agree that forgiveness is a complex construct (Enright & Fitzgibbons, 2000). It involves cognitive (Flanigan, 1992; Gordon & Baucom, 1998; Thompson et al., 2005), affective (Worthington & Scherer, 2004), motivational (McCullough et al., 1997), decisional (DiBlasio, 1998), and interpersonal (e.g., Baumeister et al., 1998) aspects. It is usually defined as a process of reducing negative thoughts, feelings, and behaviors toward a transgressor (Enright, 1996; McCullough et al., 1998; Rye & Pargament, 2002) which might be accompanied by evoking positive thoughts, feelings, and actions toward the harm-doer (Sells & Hargrave, 1998; Subkoviak et al., 1995). Thus, forgiveness occurs via shift in beliefs, affects, and motives (see Toussaint & Friedman, 2009; Fehr et al., 2010).

Forgiveness conceptualized in this manner might be viewed not only as a single act following a particular offense, but also as a dimension of personality (Hill & Allemand, 2010; Kamat, et al., 2006). The latter (also referred to as *forgivingness*; Roberts, 1995) can be defined as one’s tendency to forgive regardless of time, relationships, and situations (Berry et al., 2001; Brown, 2003) and is mainly related to other personality traits, such as agreeableness (Berry et al., 2001; Kamat et al., 2006), neuroticism (Allemand et al., 2008; Maltby et al., 2004), religiousness (Brown et al., 2007; Edwards et al., 2002), empathy (Brown, 2003; Chung, 2014; Giammarco & Vernon, 2014), gratitude (McCullough et al., 2002; Touissant & Friedman, 2009), cognitive flexibility (Thompson et al., 2005), rumination (Berry et al., 2005; Burnette et al., 2009; Chung, 2014), or narcissism (Brown, 2004; Eaton et al., 2006; Fatfouta et al., 2015). Many of these personality characteristics are usually gender-specific, which suggests that dispositional forgiveness might also differ depending on gender. A similar suggestion might be drawn from the previous meta-analytic reviews (Miller & Worthington, 2015; Miller et al., 2008).

However, the results of studies comparing dispositional forgiveness in men and women are ambiguous. For instance, Ka et al. (2006) found that women yielded significantly higher scores on the Forgiving Personality Scale. Also, Mullet et al. (1998) found that women, more than men, were prone to forgive rather than to seek revenge. Toussaint et al. (2008) showed forgiveness of others, feeling forgiven by God and seeking forgiveness to be greater among females than males. In turn, Brown (2003) revealed that women scored lower than men in the tendency to forgive*.* Moreover*,* studies have reported that the observed relationships between different variables and forgivingness vary notably as a function of the participants' genders. Neto and Mullet (2004) showed that the relationship between self-esteem and propensity to forgive was positive in men, but negative among women. Ryan and Kumar (2005) found willingness to forgive significantly correlated with anxiety and symptoms severity in males, but not in females. Toussaint et al. (2008) showed that for women, high levels of forgiveness of others, forgiveness of self, feeling forgiven by God, and seeking forgiveness significantly reduced the odds of major depressive symptoms, while for men, only forgiveness of self was a significant predictor of depression. On the other hand, several studies (Berry et al., 2001; Brose et al., 2005; Cohen et al., 2006) found no gender differences in disposition to forgive. Thus, gender differences in forgivingness deserve to be more directly addressed (Miller & Worthington, 2015), the more so that the existing literature provides theoretical and empirical framework for putting forward specific hypotheses. They might be derived from concepts and research on interpersonal orientation and affective traits (cf. Miller et al., 2008; Toussaint et al., 2008).

### Conceptual Framework

Many gender differences in cognition, motivation, emotion, and social behavior may be explained in terms of men's and women's different self-construals (Markus & Kitayama, 1991). Male self-construals are typically more independent, autonomous, distinct, and separated from others, while female self-construals are more interdependent, connected, and less differentiated from others (Agerström et al., 2006; Cross & Madson, 1997). Such differences in defining oneself may have an impact on gender differences in responding to and dealing with transgressions. Women are likely to be more motivated to forgive because forgiveness helps them maintain a connection with others (Markus & Kitayama, 1991) by pushing people to reconciliation (Worthington et al., 2019). The link between interdependence and forgivingness was found to be notably stronger among women than men (Neto & Mullet, 2004). It might be particularly important when female stress and detachment following interpersonal transgressions are intensive and an individual needs to cope with them. Although there are different ways to reduce such distress (Wade & Worthington, 2003), only forgiveness results in pro-social acting and relating with others (Worthington & Wade, 1999). In confirmation, Neto and Mullet (2004) showed that loneliness was positively associated with propensity to forgive among women, whereas it was negatively correlated in men (Neto & Mullet, 2004).

Gender and different self-construals might not only affect the level of forgiveness, but they may also play a role in processing forgiveness, especially in the relationship between emotions and forgiveness. Different self-construals bring significant consequences for experiencing or/and suppressing emotions, both positive and negative (Markus & Kitayama, 1991). For instance, holding an interdependent view of the self inhibits overly expressed anger or frustration, which are emotional states closely related to forgiveness. Consequently, links between the tendency to experience and control different emotions and the ability to forgive might differ in men and women. Generally, negative affect and emotions such as anger, shame, and guilt may impede the process of forgiveness (Hall & Fincham, 2008; Macaskill, 2012; Mróz & Kaleta, 2017), and less forgiving individuals tend to experience higher levels of bitterness, hostility, fear, anxiety, or depressive affect (Barcaccia et al., 2020; Berry et al., 2005; Rye et al., 2001; Toussaint et al., 2008; Lawler-Row & Piferi, 2006; Burnette et al., 2009). In turn, positive emotions, like sympathy, compassion, or happiness, increase the likelihood of forgiving (Baker et al., 2017; Uysal & Satici, 2014). Still, there are gender differences in various affective traits. For instance, women often score higher in negative affect, especially for anxiety and depression (Fujita et al., 1991; Parker & Brotchie, 2010; Thomsen et al., 2005). In addition, emotion regulation which affects emotional experience and expression (Baker et al., 2017; Gross, 1998; Gross & John, 2003; Thomsen et al., 2005) and which is related to forgiveness (Baker et al., 2017; Hodgson & Wertheim, 2007; Mami et al., 2019; McCullough et al., 2001; Witvliet et al., 2010, 2011, 2015), might be gender-related (Gross & John, 2003; Koch et al., 2007; Matud, 2004; McRae et al., 2008; Thomsen et al., 2005). Summing up, our reasoning points to gender differentiation in forgivingness and its associations with emotional variables. Thus, the first goal of the present study was to examine gender differences in the levels of dispositional forgiveness. We put forward the hypothesis that women would score higher in multiple dimensions of forgivingness (H1). The second goal was to examine how emotional variables are associated with different aspects of dispositional forgiveness among women and men. We expected that negative affect, anxiety and control of anger, of depression and of anxiety, would be significantly and inversely correlated with forgivingness, whereas positive affect positively correlated with the ability to forgive in both genders. However, as shown, the link between emotionality and dispositional forgiveness may vary across genders in terms of direction or magnitude. Thus, we hypothesized the moderating role of gender in the analyzed relationships. From the self-construal approach positive emotions might motivate females to be more forgiving than men, whereas the experience of negative emotions may make women less reluctant than men to forgive in order to find peace after transgressions. Consequently, we hypothesized that the relationship between positive affect and dispositional forgiveness would be more positive for females (H2), while associations between negative affect and forgiveness, anxiety and forgiveness, and between suppression of emotions and forgiveness would be less negative for them (H3).

## Method

### Participants

Data were collected from 638 adults, 481 women (75.4%), and 148 men (23.2%). Nine participants did not specify their gender. They were from 19 to 69 years of age, with the mean age of 33.45 (SD = 11.81). The majority of respondents were married (49%), 1.7% widowed, 4.3% divorced, and 45% never married. Educational attainment ranged from vocational education (21.1%), through secondary (24.8%), and college (14.8%) to higher education (39.3%). Finally, 48.4% of the respondents lived in the country, 16.1% in towns, and 35.4% in cities.

### Measures

#### Forgivingness

Disposition to forgive was measured using the Polish version (Kaleta et al., 2016) of the Heartland Forgiveness Scale (Thompson & Snyder, 2003; Thompson et al., 2005). HFS is a multidimensional tool assessing dispositional forgiveness of self, others, and situations beyond anyone's control. Participants rate their responses to 18 items on a 7-point scale. The Polish version consists of two scales that allow to measure forgivingness in two separate domains—positive (P-scale; benevolent thoughts, feelings, and behaviors) and negative (N-scale; reduction of hostile thoughts, feelings, and behaviors), and six subscales with the distinction of forgiveness of self, others, and situations (P-self, P-others, P-situations, N-self, N-others, N-situations). Higher scores on each scale reflect a higher level of forgivingness in every domain. The total HFS score indicates how forgiving a person tends to be. Sample items include “Although I feel badly at first when I mess up, over time I can give myself some slack” and “If others mistreat me, I continue to think badly of them.” Cronbach's alpha (internal consistency) values were found as follows: for overall HFS 0.76, for P-scale 0.70, and for N-scale 0.81, and from 0.50 to 0.81 for subscales (Kaleta et al., 2016).

#### Positive and Negative Affect

Positive–negative affect was measured using the Polish version (SUPIN C30; Brzozowski, 2010) of the Positive and Negative Affect Schedule (PANAS; Watson et al., 1988). The scale consists of 30 items, 15 items for positive affect (from 15 to 75 points) and 15 items for negative affect (from 15 to 75 points). We focused on measuring affect at a trait level. Using a 5-point scale, the participants are asked to indicate the degree to which they usually feel each emotion, e.g., ashamed, irritated, afraid, exited, proud, active. The higher the score, the higher the level of particular affectivity. Cronbach's alpha for PANAS ranged from 0.73 to 0.95 (Brzozowski, 2010).

#### Anxiety

Anxiety was measured using the State-Trait Anxiety Inventory (STAI, Spielberger et al., 1983), more specifically the Polish version of the tool (Wrześniewski et al., 2002). This is a widely used, valid instrument. In the present study, participants rated 20 trait anxiety items (STAI-X2) assessing how an individual generally feels, using a 4-point Likert scale. Sample items: "I worry too much over something that really doesn’t matter," “I am a steady person.” The score range is from 20 (low) to 80 (high anxiety). Cronbach's alpha measuring internal consistency of the Polish version ranged from 0.76 to 0.92 in the group of adult women and men.

#### Emotional control

Emotional control was measured using the Courtauld Emotional Control Scale (CECS) developed by Watson and Greer (1983) and adapted in Poland by Juczyński (2009). The questionnaire allows to evaluate the extent to which individuals report suppression as an emotion regulation strategy in difficult situations. The CECS consists of 21 items divided into three subscales, each of which contains seven statements concerning the way of suppressing or expressing anger, depression and anxiety (from 7 to 28 points in every subscale). Participants are asked to respond to the phrases “When I feel angry…”, “When I feel anxious (worried)…”, and “When I feel unhappy (miserable)…” with statements such as “I keep quiet,” “I bottle it up,” or “I tell others about it” rated on a 4-point scale. The total emotional control index is established by summing up the results of the three subscales. The higher the result, the more negative emotions are suppressed. Reliability of the Polish version (Cronbach's alpha) is: for the control of anger 0.80, depression 0.77, anxiety 0.78, and for the total emotional control 0.87 (Juczyński, 2009).

### Statistical methods

First, we calculated descriptive statistics (means and standard deviations) for all the variables in women and men separately. Due to unequal sample sizes we used the Mann–Whitney U test to examine gender differences and the Glass’*Δ* estimate to evaluate the effect size. Second, correlational analyses were performed to explore relationships among emotional variables and multidimensional forgiveness in females and males. Finally, to test whether gender moderated the relationship between emotionality and disposition to forgive, we used a set of moderation analyses using the Process macro for SPSS (Model 1, version 3.5, Hayes, 2018). Moderation models have been proposed with positive–negative affect, anxiety, emotional control (overall and control of anger, of anxiety, and of depression) as predictors, dimensions of forgivingness as outcome variables, and gender as a moderator. In order to properly estimate interactions of emotional variables and gender on forgivingness, mean centering and standardizing were applied. Bias-corrected confidence intervals (CI 95%) and the bootstrapping procedure (samples = 5000) were used to calculate direct and indirect effects.

## Results

Table 1 presents gender differences in dimensions of forgivingness and analyzed variables and provides descriptive statistics, statistical significance*,* and Glass’ Δ effect size estimates.

**Table 1:** Mean levels and standard deviations of dispositional forgiveness and affective traits by gender

	Women (*n* = 476)		Men (*n* = 146)		Mann–Whitney *U*	*p*	*Glass’* Δ
	*M*	*SD*	*M*	*SD*			
Total forgiveness	81.94	12.15	84.22	11.48	30,638.00	.030	− .19
Positive forgiveness (P-scale)	44.23	6.74	43.80	6.77	33,321.00	.452	.06
P-self	15.05	2.98	14.82	3.09	33,907.00	.656	.07
P-others	14.18	3.06	13.86	3.06	32,864.00	.319	.10
P-situations	15.01	2.84	15.12	2.91	33,728.50	.509	− .04
Reduced unforgiveness (N-scale)	37.71	9.30	40.42	9.59	29,224.00	.004	− .28
N-self	12.27	4.37	13.62	4.39	28,510.50	.001	− .31
N-others	12.95	3.61	12.97	3.68	33,522.00	.542	− .01
N-situations	12.49	3.89	14.01	3.67	26,825.50	.000	− .41
Positive affect	48.07	9.35	50.45	10.49	29,247.00	.009	− .27
Negative affect	35.32	11.96	29.02	10.40	23,577.50	.000	.61
Anxiety	46.50	9.21	41.18	9.37	21,296.50	.000	.57
Emotional control – total score	50.17	10.99	53.23	9.70	27,061.50	.000	− .32
Control of anger	15.97	4.69	16.43	4.37	31,869.50	.237	− .11
Control of depression	17.27	4.35	17.84	3.76	31,098.50	.110	− .15
Control of anxiety	16.93	4.28	18.95	4.50	24,467.50	.000	− .45

Men showed higher levels of general forgivingness and more willingness to overcome unforgiveness, especially toward oneself and situations beyond anyone’s control. Gender did not differentiate the results in positive facets of the disposition to forgive. There were gender differences in affective traits. Women scored significantly higher in negative affect and anxiety. Men reported greater positive affect and emotional control, in particular the suppression of anxiety. It should be noted that according to the Glass’ effect size estimate, gender differences were rather small to moderate in magnitude. The largest differences between women and men were reported for reduced unforgiveness of situations, negative affect, anxiety, and control of anxiety.

Table 2 shows correlations (Pearson’s r) between the variables in women and men.

**Table 2:** Correlations between measures for women and men

	1	2	3	4	5	6	7	8	9	10	11	12	13	14	15	16
	**Men** (n = 146)															
Total forgiveness	–	.55^**^	.44^**^	.45^**^	.34^**^	.81^**^	.67^**^	.63^**^	.68^**^	.25^**^	− .42^**^	− .43^**^	.00	− .09	− .17^*^	.24^**^
Positive forgiveness (P-scale)	.65^**^	–	.74^**^	.75^**^	.75^**^	− .04	− .07	− .01	− .02	.24^**^	− .12	− .12	.08	.07	.00	.10
P-self	.45^**^	.72^**^	–	.32^**^	.33^**^	.01	− .02	− .01	.07	.13	− .15	− .15	− .02	.01	− .08	.01
P-others	.53^**^	.78^**^	.29^**^	–	.37^**^	.01	− .07	.11	− .02	.17^*^	− .16	− .06	.08	.04	.01	.3
P-situations	.51^**^	.78^**^	.34^**^	.46^**^	–	− .12	− .06	− .13	− .10	.24^**^	.04	− .07	.11	.11	.06	.08
Reduction of unforgiveness (N-scale)	.83^**^	.13^**^	.06	.13^**^	.10^*^	–	.85^**^	.76^**^	.83^**^	.13	− .42^**^	− .44^**^	− .05	− .16	− .20^*^	.22^**^
N-self	.63^**^	.01	.03	.00	− .00	.81^**^	–	.45^**^	.59^**^	.17^*^	− .33^**^	− .40^**^	− .03	− .21^*^	− .14	.24^**^
N-others	.59^**^	.10^*^	− .04	.20^**^	.06	.70^**^	.30^**^	–	.46^**^	.01	− .25^**^	− .22^**^	− .05	− .06	− .14	.08
N-situations	.74^**^	.20^**^	.15^**^	.12^*^	.18^**^	.83^**^	.54^**^	.40^**^	–	.13	− .44^**^	− .44^**^	− .04	− .11	− .21^*^	.19^*^
Positive affect	.30^**^	.25^**^	.17^**^	.19^**^	.21^**^	.21^**^	.14^**^	.12^**^	.23^**^	–	− .25^**^	− .42^**^	− .11	− .12	− .14	.00
Negative affect	− .45^**^	− .25^**^	− .22^**^	− .16^**^	− .20^**^	− .40^**^	− .30^**^	− .15^**^	− .50^**^	− .23^**^	–	.73^**^	− .02	.01	.10	− .13
Anxiety	− .55^**^	− .27^**^	− .20^**^	− .17^**^	− .24^**^	− .52^**^	− .40^**^	− .23^**^	− .58^**^	− .42^**^	.64^**^	–	− .04	− .06	.08	− .11
Emotional control– total score	− .21^**^	− .04	− .01	− 04	− .05	− .25^**^	− .19^**^	− .09	− .29^**^	− .10^*^	.17^**^	.20^**^	–	.77^**^	.83^**^	.71^**^
Control of anger	− .21^**^	.00	− .01	.02	− .01	− .27^**^	− .24^**^	− .08	− .31^**^	− .10^*^	.16^*^	.17^**^	.83^**^	−	.59^**^	.20^*^
Control of depression	− .26^**^	− .07	− .01	− .06	− .09	− .28^**^	− .24^**^	− .12^**^	− .30^**^	− .16^**^	.21^**^	.25^**^	.85^**^	.58^**^	–	.39^**^
Control of anxiety	− .06	− .05	.01	− .07	− .04	− .05	.01	− .02	− .10^*^	.03	.05	.06	.79^**^	.45^**^	.53^**^	–
	Women (*n* = 476)															

^**^p < .01; ^*^p < .05

As shown in Table 2, women’s negative affect and anxiety were negatively correlated with multiple dimensions of dispositional forgiveness, whereas positive affect was positively associated with all facets of forgivingness. Women’s control of emotions, including control of anger and depression, was inversely related to total forgiveness and reduced unforgiveness, but not related to positive forgiveness. Control of anxiety was negatively associated with overcoming unforgiveness of situations beyond control.

In the case of male participants, negative affect and anxiety were inversely correlated with overall forgiveness and all facets of reducing unforgiveness. Positive affect was positively related to total and positive forgiveness (especially of situations and others). Control of anger was negatively correlated with overcoming unforgiveness of self; control of depression was negatively associated with general forgivingness and decreased unforgiveness, particularly of situations. Finally, control of anxiety was positively associated with total forgiveness and reduction of unforgiveness (general, as well as of oneself and situations).

Next, we examined the moderating role of gender in the relationship between affective traits and disposition to forgive. Sixty-three models adopting subsequent affective traits as predictors and facets of forgivingness as outcomes were tested, and eight of them confirmed the moderating role of respondents’ gender. Table 3 presents interaction effects of the examined models.^1^

**Table 3:** Indirect effects of affective variables on forgivingness moderated by gender

Moderating role of gender		Indirect effects					
Predictors	Outcomes	*B*	*SE*	*∆R2*	*F*	LLCI	ULCI
Positive affect	Total forgiveness	− .114	.108	.002	1.115	− .327	.098
	Positive forgiveness (P-scale)	− .024	.062	.000	.153	− .145	.097
	P-self	− .018	.028	.001	.391	− .073	.038
	P-others	− .011	.028	.000	.154	− .067	.044
	P-situations	.005	.026	.000	.029	− .047	.056
	Reduction of unforgiveness (N-scale)	− .090	.087	.002	1.080	− .260	.080
	N-self	.003	.041	.000	.007	− .077	.083
	N-others	− .045	.034	.003	1.760	− .112	.022
	N-situations	− .048	.035	.003	1.882	− .118	.021
Negative affect	Total forgiveness	− .011	.097	.000	.012	− .200	.179
	Positive forgiveness (P-scale)	.063	.059	.002	1.141	− .053	.179
	P-self	.010	.027	.000	.149	− .042	.062
	P-others	− .006	.027	.000	.042	− .059	.048
	P-situations	.058	.025	.009	5.339	.009	.108
	Reduction of unforgiveness (N-scale)	− .074	.077	.001	.911	− .225	.078
	N-self	− .035	.038	.001	.845	− .108	.039
	N-others	− .044	.032	.003	1.874	− .107	.019
	N-situations	.005	.030	.000	.026	− .054	.064
Anxiety	Total forgiveness	.187	.108	.004	2.997	− .025	.399
	Positive forgiveness (P-scale)	.105	.069	.004	2.306	− .031	.240
	P-self	.017	.031	.001	.287	− .044	.078
	P-others	.036	.032	.002	1.297	− .026	.098
	P-situations	.052	.029	.005	3.147	− .006	.109
	Reduction of unforgiveness (N-scale)	.082	.086	.001	.916	− .087	.251
	N-self	.000	.042	.000	.000	− .083	.083
	N-others	.005	.037	.000	.018	− .068	.078
	N-situations	.077	.034	.006	5.201	.011	.144
Emotional control – total score	Total forgiveness	.244	.113	.007	4.634	.021	.466
	Positive forgiveness (P-scale)	.081	.065	.003	1.569	− .046	.209
	P-self	− .004	.029	.000	.020	− .061	.053
	P-others	.039	.029	.003	1.746	− .019	.097
	P-situations	.047	.027	.005	2.919	− .007	.100
	Reduction of unforgiveness (N-scale)	.162	.088	.005	3.420	− .010	.335
	N-self	.063	.042	.004	2.291	− .019	.144
	N-others	.013	.034	.000	.145	− .054	.081
	N-situations	.087	.036	.009	5.947	.017	.156
Control of anger	Total forgiveness	.293	.254	.002	1.333	− .206	.792
	Positive forgiveness (P-scale)	.109	.146	.001	.557	− .178	.395
	P-self	.016	.065	.000	.057	− .112	.143
	P-others	.016	.066	.000	.059	− .114	.146
	P-situations	.077	.061	.003	1.588	− .043	.198
	Reduction of unforgiveness (N-scale)	.184	.195	.002	.891	− .199	.568
	N-self	.018	.092	.000	.037	− .163	.198
	N-others	.007	.077	.000	.008	− .145	.159
	N-situations	.161	.079	.006	4.130	.005	.317
Control of depression	Total forgiveness	.208	.287	.001	.526	− .355	.770
	Positive forgiveness (P-scale)	.101	.166	.001	.368	− .226	.427
	P-self	− .055	.074	.001	.557	− .201	.090
	P-others	.055	.075	.001	.538	− .093	.203
	P-situations	.101	.0702	.003	2.091	− .036	.238
	Reduction of unforgiveness (N-scale)	.107	.222	.000	.233	− .329	.543
	N-self	.077	.105	.001	.539	− .129	.284
	N-others	− .033	.088	.000	.141	− .205	.139
	N-situations	.063	.090	.001	.494	− .114	.241
Control of anxiety	Total forgiveness	.787	.257	.015	9.343	.281	1.292
	Positive forgiveness (P-scale)	.225	.146	.004	2.365	− .062	.512
	P-self	.004	.065	.000	.004	− .124	.132
	P-others	.140	.066	.007	4.521	− .011	.270
	P-situations	.081	.062	.003	1.713	− .040	.201
	Reduction of unforgiveness (N-scale)	.562	.201	.013	7.827	.168	.957
	N-self	.230	.094	.010	5.973	.045	.415
	N-others	.086	.078	.002	1.231	− .066	.239
	N-situations	.246	.082	.014	8.966	.085	.407

The analysis revealed no significant interaction effect of positive affect and gender on any dimension of forgiveness, but confirmed a significant effect of negative affect and gender in relation to positive forgiveness of situations (Table 3). It showed that negative affect was negatively related to this type of forgiveness for women (*B* =  − 0.046, *SE* = 0.011, CI95% = [− 0.067, − 0.025]), but not for men (*B* = 0.012, *SE* = 0.023, CI95% = [− 0.033, 0.057]). Also, the link between anxiety and reduced unforgiveness of situations was more negative for females (*B* =  − 0.24, *SE* = 0.02, CI95% = [− 0.275, − 0.211]) when compared to males (*B* =  − 0.17, *SE* = 0.03, CI95% = [− 0.224, − 0.108]). Overall emotional control was negatively associated with total forgivingness in female (*B* =  − 0.24, *SE* = 0.05, CI95% = [− 0.340, − 0.141]), but not in male respondents (*B* = 0.003, *SE* = 0.10, I95% = [− 0.195, 0.202]). Similarly, overall emotional control was negatively related to reduced unforgiveness of situations in females (*B* =  − 0.10, *SE* = 0.02, CI95% = [− 0.135, − 0.072]) and not in males (*B* =  − 0.02, *SE* = 0.03, CI95% = [− 0.079, 0.045]). Control of anger was negatively associated with reduced unforgiveness of situations for females (*B* =  − 0.25, *SE* = 0.04, CI95% = [− 0.329, − 0.185]), while the link was nonsignificant for males (*β* =  − 0.10, *SE* = 0.07, CI95% = [− 0.235, 0.042]). In contrast, in men greater control of anxiety was linked to higher total forgivingness (*B* = 0.61, *SE* = 0.22, CI95% = [0.178, 1.046]), reduced unforgiveness (*B* = 0.46, *SE* = 0.17, CI95% = [0.122, 0.801]), and reduced unforgiveness of self (*B* = 0.23, *SE* = 0.09, CI95% = [0.081, 0.399]) and in women it was not significantly related to these facets of forgiveness (respectively, *B* =  − 0.17, *SE* = 0.13, CI95% = [− 0.434, 0.083], *B* =  − 0.10, *SE* = 0.10, CI95% = [− 0.302, 0.101], *B* = 0.01, *SE* = 0.05, CI95% = [− 0.085, 0.104]). The relationship between control of anxiety and reduced unforgiveness of situations was positive for males (*B* = 0.16, *SE* = 0.07, CI95% = [0.019, 0.296]), while for females it was negative (*B* =  − 0.09, *SE* = 0.04, CI95% = [− 0.171, − 0.006]). Gender did not moderate the effect of control of depression on any dimension of forgivingness.

Thus, moderation analysis confirmed gender differences in processing forgiveness of self and of situations. Higher negative affect, anxiety, emotional control, including control of anger and of anxiety, made overcoming negative responses in situations beyond control more difficult for females than for males. In contrast, greater control of anxiety helped males to be more forgiving, especially toward themselves and situations. The moderating role of gender in relation to forgivingness of others did not occur.

## Discussion

Although neglected by most researchers, gender-related differences in forgivingness (and the associated variables) should be given more empirical attention. It is significant not only to gain more insight into the forgiveness phenomenon, but also to apply effective interventions and therapeutic techniques. Our study attempts to fill in this gap as it was aimed at direct comparison of men and women in their disposition to forgive and the way it is linked to affective traits.

The first hypothesis about a stronger tendency to forgive among women was not supported. Women revealed lower forgiveness in the total score and in its negative dimension. Few studies showed a similar trend, four of which included dispositional forgiveness (Brown, 2003; Charzyńska, 2015; Exline et al., 2004; Piątek, 2011). Two other studies were focused on forgiveness of a specific offense in close relationships, showing male partners to be more forgiving toward female partners (Finkel et al., 2002; Miller & Worthington, 2010). However, they all measured forgiveness of others, while our results demonstrated that women are less able to overcome unforgiveness toward themselves and situations, but not toward other people. Also, there were no significant differences between women and men in positive facets of forgiveness. Multidimensional measures we used might have helped us investigate gender differences more accurately. To our knowledge, our study is the first to examine gender differences in terms of that many aspects of forgivingness.

Females' lower disposition to reduce unforgiveness, especially toward self and situations beyond control, might be interpreted as a function of different self-construals. An interdependent view of the self characterizes females as focused on maintaining connection to people and being less separated from the social context (Markus & Kitayama, 1991). Their own failures and wrongdoings might be seen as disturbing the harmony with relevant others and threatening their positive self-portrait (Fincham et al., 2004). Consequently, an interdependent self-construal makes females prone to feel shame (Markus & Kitayama, 1991) which is negatively related to forgiveness, especially of self (Hall & Fincham, 2005).

Consistently with previous studies (e.g., Rye et al., 2001; Thompson et al., 2005; Uysal & Satici, 2014), we found that negative affect, anxiety, control of anger, and of depression were negatively correlated with forgivingness, while positive affect was positively related to forgiveness, in both women and men. Gender significantly moderated a number of the relationships between affective traits and forgivingness, especially links involving emotional control. Few previous studies also demonstrated gender-based correlations between different variables and forgiveness. For instance, both Fincham et al. (2002) and Toussaint and Webb (2005) found that empathy is more strongly associated with forgiveness in men than in women. Fincham et al. (2002) also showed that the direct effect of attribution of responsibility on marital forgiveness proved to be stronger for wives than for husbands. Fincham et al. (2004) demonstrated that partner reports of inefficient conflict resolution were positively correlated with retaliation, a negative dimension of marital forgiveness, for husbands only.

We found—in contrast to our hypotheses—negative relationships between negative affect, anxiety, emotional control, including control of anger and of anxiety, and willingness to forgive of situations stronger for female than for male participants. For interdependent individuals, it is important to control intense experiences of negative emotions, especially anger, because it threatens their selves (Markus & Kitayama, 1991). However, in the light of our findings strong emotional control makes forgiving difficult for women. In turn, control of anxiety was positively related to forgiveness in men. For independent selves, it is important to express self and ego-focused emotions, such as anger or frustration. Controlling anxiety in men might foster such expression and, consequently, favor forgiveness. Our results suggest that women and men need different interventions in facilitating forgiveness (Miller & Worthington, 2015). Females need more self-forgiveness interventions, releasing anxiety and promoting more open expression of negative emotions in the way they are able to accept, for instance in constructive communication. Males need work on emotion regulation, especially controlling of anxiety and of anger.

### Limitations of the Study

Although the present study attempts to fill in the gap by addressing the scarcely examined question of gender differences in forgiveness, it is burdened with a number of limitations. First, there is a significant gender imbalance in the sample. It is a common problem that women tend to participate in forgiveness studies more often than men (Root & Exline, 2011; Worthington et al., 2000). We collected data only from interested individuals who were willing to participate in the study completely voluntarily (for no remuneration). The question is whether some bonuses would encourage more men to participate in the research and help minimize the imbalance. Secondly, the design of the research is cross-sectional and the statistics are merely correlational and cannot be used to draw conclusions about causality. Longitudinal studies would be more useful in exploring the causal relationships between analyzed variables more extensively. Thirdly, we did not control some important variables, such as social desirability bias which is related to forgiveness and which may moderate the link between affective traits and forgivingness (e.g., Rye et al., 2001). Finally, because the study uses measures of dispositional forgiveness, it does not in fact address actual forgiving. Consequently, the findings cannot be extended to forgiveness occurring in natural situations. Ability to forgive is just one of many more predictors of episodic forgiveness that is, in fact, more strongly related to contextual circumstances, such as severity of the wrongdoing, apology, relational closeness, and passage of time (Boon & Sulsky, 1997; Johnson et al., 2013; Koutsos et al., 2008). Thus, our findings still do not make it possible to predict whether women or men would be more willing to forgive a particular offense in a given context. Future research should apply more complex models in which both disposition to forgive and gender would mediate and moderate the relationships between different contextual factors, episodic forgiveness, and related variables. For instance, the relationship between apology and actual forgiveness could be mediated by forgivingness and moderated by gender.

## Footnotes

1 As age is one of the key sociodemographic predictors of forgiveness (Cheng & Yim, 2008; Kaleta & Mróz, 2018), we alternatively tested each of the models after including participants' age as a covariate. Although age was a significant covariate in majority models, the findings were essentially the same as reported here.

## References

[CR1] Agerström J, Möller K, Archer T (2006). Moral reasoning: The influence of affective personality, dilemma content and gender. Social Behavior and Personality.

[CR2] Allemand M (2008). Age differences in forgivingness: The role of future time perspective. Journal of Research in Personality.

[CR3] Baker JC, Williams JK, Witvliet CVO, Hill PC (2017). Positive reappraisals after an offense: Event-related potentials and emotional effects of benefit-finding and compassion. The Journal of Positive Psychology.

[CR4] Barcaccia B, Salvati M, Pallini S, Saliani AM, Baiocco R, Vecchio GM (2020). The bitter taste of revenge: Negative affect, depression and anxiety. Current Psychology.

[CR5] Baumeister RF, Exline JJ, Sommer KL, Worthington EL (1998). The victim role, grudge theory, and two dimensions of forgiveness. Dimensions of forgiveness: Psychological research and theological perspectives.

[CR6] Berry JW, Worthington EL, Parrott L, O’Connor LE, Wade NG (2001). Dispositional forgivingness: Development and construct validity of the transgression narrative test of forgivingness (TNTF). Personality and Social Psychology Bulletin.

[CR7] Berry JW, Worthington EL, O'Connor LE, Parrott L, Wade NG (2005). Forgivingness, vengeful rumination, and affective traits. Journal of Personality.

[CR8] Boon SD, Sulsky LM (1997). Attributions of blame and forgiveness in romantic relationships: A policy-capturing study. Journal of Social Behavior and Personality.

[CR9] Brose LA, Rye MS, Lutz-Zois C, Ross SR (2005). Forgiveness and personality traits. Personality and Individual Differences.

[CR10] Brown RP (2003). Measuring individual differences in the tendency to forgive: Construct validity and links with depression. Personality and Social Psychology Bulletin.

[CR11] Brown RP (2004). Vengeance is mine: Narcissism, vengeance, and the tendency to forgive. Journal of Research in Personality.

[CR12] Brown RP, Barnes CD, Campbell NJ (2007). Fundamentalism and forgiveness. Personality and Individual Differences.

[CR13] Brzozowski, P. (2010). Skala uczuć pozytywnych i negatywnych SUPIN. *Polska adaptacja PANAS Davida Watsona i Lee Anny Clark. Podręcznik [Scale of positive and negative affects SUPIN: Polish adaptation of PANAS by David Watson & Anna Clark]*. Warsaw: PTP.

[CR14] Burnette JL, Davis DE, Green JD, Worthington EL, Bradfield E (2009). Insecure attachment and depressive symptoms: The mediating role of rumination, empathy, and forgiveness. Personality and Individual Differences.

[CR15] Charzyńska E (2015). Sex differences in spiritual coping, forgiveness, and gratitude before and after a basic alcohol addiction treatment program. Journal of Religion and Health.

[CR16] Cheng ST, Yim YK (2008). Age differences in forgiveness: The role of future time perspective. Psychology of Aging.

[CR17] Chung MS (2014). Pathways between attachment and marital satisfaction: The mediating roles of rumination, empathy, and forgiveness. Personality and Individual Differences.

[CR18] Cohen AB, Malka A, Rozin P, Cherfas L (2006). Religion and unforgivable offenses. Journal of Personality.

[CR19] Cross SE, Madson L (1997). Models of the self: Self-construals and gender. Psychological Bulletin.

[CR20] DiBlasio FA (1998). The use of a decision-based forgiveness intervention within intergenerational family therapy. Journal of Family Therapy.

[CR21] Eaton J, Struthers CW, Santelli AG (2006). Dispositional and state forgiveness: The role of self-esteem, need for structure, and narcissism. Personality and Individual Differences.

[CR22] Edwards LM, Lapp-Rincker RH, Magyar-Moe JL, Rehfeldt JD, Ryder JA, Brown JC, Lopez SJ (2002). A positive relationship between religious faith and forgiveness: Faith in the absence of data?. Pastoral Psychology.

[CR23] Enright RD (1996). Counseling within the forgiveness triad: On forgiving, receiving forgiveness, and self-forgiveness. Counseling and Values.

[CR24] Enright RD, Fitzgibbons RP (2000). Helping clients forgive: An empirical guide for resolving anger and restoring hope.

[CR25] Exline JJ, Baumeister RF, Bushman BJ, Campbell WK, Finkel EJ (2004). Too proud to let go: Narcissistic entitlement as a barrier to forgiveness. Journal of Personality and Social Psychology.

[CR26] Fatfouta R, Gerlach TM, Schröder-Abé M, Merkl A (2015). Narcissism and lack of interpersonal forgiveness: The mediating role of state anger, state rumination, and state empathy. Personality and Individual Differences.

[CR27] Fehr R, Gelfand MJ, Nag M (2010). The road to forgiveness: A meta-analytic synthesis of its situational and dispositional correlates. Psychological Bulletin.

[CR28] Fincham FD, Beach SR, Davila J (2004). Forgiveness and conflict resolution in marriage. Journal of Family Psychology.

[CR29] Fincham FD, Paleari FG, Regalia C (2002). Forgiveness in marriage: The role of relationship quality, attributions, and empathy. Personal Relationships.

[CR30] Finkel EJ, Rusbult CE, Kumashiro M, Hannon PA (2002). Dealing with betrayal in close relationships: Does commitment promote forgiveness?. Journal of Personality and Social Psychology.

[CR31] Flanigan B (1992). Forgiving the unforgivable.

[CR32] Fujita F, Diener E, Sandvik E (1991). Gender differences in negative affect and well-being: The case for emotional intensity. Journal of Personality and Social Psychology.

[CR33] Giammarco EA, Vernon PA (2014). Vengeance and the dark triad: The role of empathy and perspective taking in trait forgivingness. Personality and Individual Differences.

[CR92] Gordon KC, Baucom DH (1998). Understanding betrayals in marriage: A synthesized model of forgiveness. Family Process.

[CR34] Gordon KC, Baucom DH, Snyder DK, Worthington E (2005). Forgiveness in couples: Divorce, infidelity, and couples therapy. Handbook of forgiveness.

[CR35] Gross JJ (1998). The emerging field of emotion regulation: An integrative review. Review of General Psychology.

[CR36] Gross JJ, John OP (2003). Individual differences in two emotion regulation processes: Implications for affect, relationships, and well-being. Journal of Personality and Social Psychology.

[CR37] Hall JH, Fincham FD (2008). The temporal course of self–forgiveness. Journal of Social and Clinical Psychology.

[CR38] Hayes AF (2018). Introduction to mediation, moderation, and conditional process analysis: A regression-based approach.

[CR39] Hill PL, Allemand M (2010). Forgivingness and adult patterns of individual differences in environmental mastery and personal growth. Journal of Research in Personality.

[CR40] Hodgson LK, Wertheim EH (2007). Does good emotion management aid forgiving? Multiple dimensions of empathy, emotion management and forgiveness of self and others. Journal of Social and Personal Relationships.

[CR41] Johnson HD, Wernli MA, LaVoie JC (2013). Situational, interpersonal, and intrapersonal characteristic associations with adolescent conflict forgiveness. The Journal of Genetic Psychology.

[CR42] Juczyński, Z. (2012). *Narzędzia pomiaru w promocji i psychologii zdrowia [Measuring tool in promotion and health psychology]*. Warsaw: PTP.

[CR43] Kaleta K, Mróz J (2018). Forgiveness and life satisfaction across different age groups in adults. Personality and Individual Differences.

[CR44] Kaleta K, Mróz J, Guzewicz M (2016). Polska adaptacja Skali Przebaczenia – Heartland Forgiveness Scale [Polish adaptation of the Heartland Forgiveness Scale]. Przegląd Psychologiczny.

[CR45] Kamat VI, Jones WH, Lawler-Row KA (2006). Assessing forgiveness as a dimension of personality. Individual Differences Research.

[CR46] Koch K, Pauly K, Kellermann T, Seiferth NY, Reske M, Backes V, Schneider F (2007). Gender differences in the cognitive control of emotion: An fMRI study. Neuropsychologia.

[CR47] Koutsos P, Wertheim EH, Kornblum J (2008). Paths to interpersonal forgiveness: The roles of personality, disposition to forgive and contextual factors in predicting forgiveness following a specific offence. Personality and Individual Differences.

[CR48] Lawler-Row KA, Piferi RL (2006). The forgiving personality: Describing a life well lived?. Personality and Individual Differences.

[CR49] Macaskill A (2012). Differentiating dispositional self- forgiveness from other-forgiveness: Associations with mental health and life satisfaction. Journal of Social and Clinical Psychology.

[CR50] Maltby J, Day L, Barber L (2004). Forgiveness and mental health variables: Interpreting the relationship using an adaptational-continuum model of personality and coping. Personality and Individual Differences.

[CR51] Mami S, Gholami M, Ahmadi V (2019). Investigating the simple and multiple correlation of emotional regulation with marital forgiveness and family efficacy in married female students of Islamic Azad University of Ilam. Journal of Basic Research in Medical Sciences.

[CR52] Markus H, Kitayama S (1991). Culture and the self: Implications for cognition, emotion, and motivation. Psychological Review.

[CR53] Matud MP (2004). Gender differences in stress and coping styles. Personality and Individual Differences.

[CR54] McCullough ME, Bellah CG, Kilpatrick SD, Johnson JL (2001). Vengefulness: Relationships with forgiveness, rumination, well-being, and the big five. Personality and Social Psychology Bulletin.

[CR55] McCullough ME, Emmons RA, Tsang JA (2002). The grateful disposition: A conceptual and empirical topography. Journal of Personality and Social Psychology.

[CR56] McCullough ME, Rachal KC, Sandage SJ, Worthington EL, Brown SW, Hight TL (1998). Interpersonal forgiving in close relationships: II. Theoretical elaboration and measurement. Journal of Personality and Social Psychology.

[CR57] McCullough ME, Worthington EL, Rachal KC (1997). Interpersonal forgiving in close relationships. Journal of Personality and Social Psychology.

[CR58] McRae K, Ochsner KN, Mauss IB, Gabrieli JJ, Gross JJ (2008). Gender differences in emotion regulation: An fMRI study of cognitive reappraisal. Group Processes & Intergroup Relations.

[CR59] Miller AJ, Worthington EL, McDaniel MA (2008). Gender and forgiveness: A meta–analytic review and research agenda. Journal of Social and Clinical Psychology.

[CR60] Miller AJ, Worthington EL (2010). Sex differences in forgiveness and mental health in recently married couples. The Journal of Positive Psychology.

[CR61] Miller AJ, Worthington EL, Toussaint LL, Worthington EL, Williams DR (2015). Sex, forgiveness, and health. Forgiveness and health.

[CR62] Mróz J, Kaleta K (2017). Cognitive and emotional predictors of episodic and dispositional forgiveness. Polish Psychological Bulletin.

[CR95] Mullet E, Houdbine A, Laumonier S, Girard M (1998). “Forgivingness”: Factor structure in a sample of young, middle-aged, and elderly adults. European Psychologist.

[CR63] Neto F, Mullet E (2004). Personality, self-esteem, and self-construal as correlates of forgivingness. European Journal of Personality.

[CR64] Parker G, Brotchie H (2010). Gender differences in depression. International Review of Psychiatry.

[CR65] Piątek M (2011). Przebaczenie w doświadczeniu życiowym kobiet i mężczyzn [Forgiveness in the life experience of women and men]. Nowiny Lekarskie.

[CR66] Roberts RC (1995). Forgivingness. American Philosophical Quarterly.

[CR67] Root BL, Exline JJ (2011). Gender differences in response to experimental forgiveness prompts: Do men show stronger responses than women?. Basic and Applied Social Psychology.

[CR68] Ryan RB, Kumar VK (2005). Willingness to forgive: Relationships with mood, anxiety and severity of symptoms. Mental Health, Religion & Culture.

[CR69] Rye MS, Loiacono DM, Folck CD, Olszewski BT, Heim TA, Madia BP (2001). Evaluation of the psychometric properties of two forgiveness scales. Current Psychology.

[CR70] Rye MS, Pargament KI (2002). Forgiveness and romantic relationships in college: Can it heal the wounded heart?. Journal of Clinical Psychology.

[CR71] Sells JN, Hargrave TD (1998). Forgiveness: A review of the theoretical and empirical literature. Journal of Family Therapy.

[CR72] Spielberger CD, Gorsuch RL, Lushene R, Vagg PR, Jacobs GA (1983). Manual for the state-trait anxiety inventory.

[CR73] Subkoviak MJ, Enright RD, Wu CR, Gassin EA, Freedman S, Olson LM (1995). Measuring interpersonal forgiveness in late adolescence and middle adulthood. Journal of Adolescence.

[CR74] Thompson LY, Synder CR, Lopez SJ, Snyder CR (2003). Measuring forgiveness. Positive psychological assessment: A handbook of models and measures.

[CR75] Thompson LY, Snyder CR, Hoffman L, Michael ST, Rasmussen HN, Billings LS, Heinze L, Neufeld JE, Shorey HS, Roberts JC, Roberts DE (2005). Dispositional forgiveness of self, others, and situations. Journal of Personality.

[CR76] Thomsen DK, Mehlsen MY, Viidik A, Sommerlund B, Zachariae R (2005). Age and gender differences in negative affect—Is there a role for emotion regulation?. Personality and Individual Differences.

[CR77] Toussaint L, Friedman P (2009). Forgiveness, gratitude, and well-being: The mediating role of affect and beliefs. Journal of Happiness Studies.

[CR78] Toussaint L, Webb JR (2005). Gender differences in the relationship between empathy and forgiveness. The Journal of Social Psychology.

[CR79] Toussaint LL, Williams DR, Musick MA, Everson-Rose SA (2008). The association of forgiveness and 12-month prevalence of major depressive episode: Gender differences in a probability sample of US adults. Mental Health, Religion and Culture.

[CR80] Uysal R, Satici SA (2014). The mediating and moderating role of subjective happiness in the relationship between vengeance and forgiveness. Educational Sciences: Theory and Practice.

[CR81] Wade NG, Worthington EL (2003). Overcoming interpersonal offenses: Is forgiveness the only way to deal with unforgiveness?. Journal of Counseling and Development.

[CR82] Watson D, Clark LA, Tellegen A (1988). Development and validation of brief measures of positive and negative affect: The PANAS scales. Journal of Personality and Social Psychology.

[CR83] Watson M, Greer S (1983). Development of a questionnaire measure of emotional control. Journal of Psychosomatic Research.

[CR84] Witvliet CVO, DeYoung N, Hofelich A, DeYoung P (2011). Compassionate reappraisal and emotion suppression as alternatives to offense-focused rumination: Implications for forgiveness and psychophysiological well-being. The Journal of Positive Psychology.

[CR85] Witvliet CVO, Hofelich Mohr AJ, Hinman NG, Knoll RW (2015). Transforming or restraining rumination: The impact of compassionate reappraisal versus emotion suppression on empathy, forgiveness, and affective psychophysiology. The Journal of Positive Psychology.

[CR86] Witvliet CVO, Knoll RW, Hinman NG, DeYoung PA (2010). Compassion-focused reappraisal, benefit-focused reappraisal, and rumination after an interpersonal offense: Emotion-regulation implications for subjective emotion, linguistic responses, and physiology. The Journal of Positive Psychology.

[CR87] Worthington EL, Rueger SY, Davis EB, Wortham J (2019). “Mere” Christian forgiveness: An ecumenical Christian conceptualization of forgiveness through the lens of stress-and-coping theory. Religions.

[CR88] Worthington EL, Sandage SJ, Berry JW, McCullough ME, Pargament KI, Thoresen CE (2000). Group interventions to promote forgiveness. Forgiveness: Theory, research, and practice.

[CR89] Worthington EL, Scherer M (2004). Forgiveness is an emotion-focused coping strategy that can reduce health risks and promote health resilience: Theory, review, and hypotheses. Psychology & Health.

[CR90] Worthington EL, Wade NG (1999). The psychology of unforgiveness and forgiveness and implications for clinical practice. Journal of Social and Clinical Psychology.

[CR91] Wrześniewski, K., Sosnowski, T., & Matusik, D. (2002). *Inwentarz Stanu i Cechy Lęku STAI. Polska adaptacja STAI. Podręcznik. (State-Trait Anxiety Inventory manual – Polish adaptation)*. Warsaw: PTP.

